# Statistical regularities in natural scenes that support figure-ground segregation by neural populations

**DOI:** 10.1371/journal.pcbi.1013573

**Published:** 2025-10-17

**Authors:** Clara T. Friedman, Minqi Wang, Thomas Yerxa, Bryce A. Arseneau, Xin Huang, Emily A. Cooper

**Affiliations:** 1 Herbert Wertheim School of Optometry & Vision Science, University of California, Berkeley, Berkeley, California, United States of America; 2 Center for Neural Science, New York University, New York City, New York, United States of America; 3 Department of Neuroscience, University of Wisconsin, Madison, Madison, Wisconsin, United States of America; 4 Helen Wills Neuroscience Institute, University of California, Berkeley, Berkeley, California, United States of America; Soochow University, CHINA

## Abstract

Differentiating objects, people, and animals from their surroundings is a key visual function, referred to as figure-ground segregation. Psychological research has established that humans use diverse visual features such as shape, texture, motion, and distance to identify figures. However, our understanding of the neural computations supporting figure-ground segregation remains incomplete. Recent neurophysiological observations in cortical area MT of primates – a region important for motion and depth processing – suggest that neurons in this area *favor* visual features that intuitively map onto figures, such as faster motion and closer distances. Inspired by these new observations, here we test the hypothesis that figures in natural scenes contain statistical regularities in motion and distance detectable at the scale of neuronal receptive fields. We combined statistical measurements of motion and distance from natural scenes with figure-ground annotations and simulations of receptive field inputs. Within simulated receptive fields, inputs corresponding to figures tended to move faster and more coherently, and tended to be nearer in distance, than the surrounding area. Our simulation predicts that the statistical regularities associated with figure motion increase notably with retinal eccentricity, while the distance statistics do not. Lastly, we implemented a simple neural population model illustrating how MT response properties, in combination with these statistics, can favor the representation of visual features associated with figures. These results enrich our understanding of the computations supporting figure-ground segregation, provide a normative account for recent neurophysiological observations, and contribute to converging lines of evidence that the brain exploits natural statistics to prioritize behaviorally-relevant information.

## Author summary

For many visually-guided activities, animals must first segment out relevant objects, people, and animals from the surroundings. This process is called figure-ground segregation. We tested the hypothesis that recently discovered neuronal response properties reflect a neural code that prioritizes visual features typical of figures in natural scenes – specifically, their motion and distance characteristics. Our approach employed natural scene statistics, precise image annotations, and behavioral/neural simulations. The findings support our working hypothesis, make new and testable predictions for neurophysiology, and illustrate the diverse ways that the statistics of natural stimuli can support not only efficient neural codes, but also neural codes that boost behaviorally-relevant information for tasks like figure-ground segregation.

## Introduction

Figure-ground segregation is critical for the visual perception of natural scenes, for guiding eye movements, and for allocating attention [1,2,3,4,5]. Figure-ground segregation is also a computationally demanding problem, requiring the observer to differentiate the visual features associated with “figures” (e.g., objects, people, animals) from the surrounding “ground” areas in cluttered natural scenes. Perceptual research suggests that shape, texture, motion, and distance are all used to differentiate and identify figures [6]. It has long been thought that these visual cues for perceptual organization reflect statistical regularities in the natural world [7]. That is, visual features associated with figure perception should be features that figures are most likely to have in nature. Indeed, as large-scale measurements of visual scene statistics have become feasible, studies have repeatedly shown that well-known perceptual organization cues have good ecological validity [8,9,10]. However, our understanding of the neural computations that support figure-ground segregation is still developing. In this report, we use two visual features – motion and distance – as a test case to jointly investigate natural visual statistics of figures and potential neural coding schemes supporting figure-ground segregation.

For decades, researchers have proposed that neural populations in mid-level brain areas play key roles in figure-ground segregation via their selectivity for specific visual features. For example, when figures are demarcated by textural cues, V4 has been proposed as a hub supporting figure-ground segregation as well as cortico-cortical feedback to enhance figure representations in V1 [11,12,13]. V4 has also been implicated in complex shape recognition through its contour selectivity [14,15]. Neurons in the middle-temporal cortex (area MT) are well-known to exhibit motion-based surround suppression and center-surround antagonism, which may support the segmentation of moving figures [16,17,18,19]. Neurons in V4, MT and beyond are also selective for object distance via tuning for binocular disparity, and depth edges are known to be an important perceptual cue in figure-ground segregation [20,21,22,23,24,25].

Several recent observations about response properties in area MT of macaque monkeys raise a compelling new hypothesis about how neural populations support figure-ground segregation. These observations come from studies that stimulate the same MT neuron with multiple surfaces, similar to what occurs at a figure-ground border. For example, Huang and colleagues characterized MT neurons’ speed tuning curves for a single moving stimulus, and then measured responses to two stimuli moving simultaneously at different speeds within the same receptive field [26,27]. For a range of speeds, neuronal responses to these bi-speed stimuli were biased towards the responses elicited by the faster moving stimulus alone. Similar MT response biases towards stimuli with more coherent motion have been previously reported [28]. And while measured MT responses when stimulated by multiple surfaces at different distances are more varied [29,30], there is evidence that these neurons overall prefer near distances when stimulated by single surfaces [31]   . It stands to reason that if figures in natural environments tend to move faster and more coherently than their surroundings, and to be nearer in distance, these neuronal response patterns might support figure-ground segregation by prioritizing the representation of visual figures, particularly when receptive fields capture a combination of figure and ground regions.

Inspired by these observations, we aimed to determine if figures in natural scenes reliably correspond to faster motion, more coherent motion, and nearer distances at spatial scales comparable to MT receptive fields. We began by accumulating datasets of motion maps and distance maps from natural scenes. We then obtained precise annotations of the visual figures in these datasets from professional human annotators. A simulation of the content falling within MT receptive fields, which incorporated both receptive field sizes and eye movements, revealed that motion and distance provide reliable patterns for differentiating figures. Lastly, a simulated population model analysis suggests that these patterns can be exploited to prioritize the representation of visual features associated with figures, even when individual neuronal receptive fields are only partially stimulated by figures.

## Materials and methods

### Motion map dataset

The motion map dataset was collected de nuevo for this project. It was designed to capture a range of real-world motion patterns in typical scenes that contain both animate and inanimate figures.

#### Recording device.

Movies were recorded with a FLIR Grasshopper3 camera (GS3-U3-23S6M) affixed with a Fujinon CF12.5HA-11” lens. The spatial resolution of the movie frames was 1024 x 1024 pixels (42 deg x 42 deg, ~ 0.04 deg/pixel) with a grayscale bit-depth of 10 bits. Movies were captured at 120Hz, with each frame saved as an uncompressed RAW file. All movies were captured outdoors and we used a fixed shutter speed of 8.25 ms. At this shutter speed, good dynamic range was achieved on sunny days with a sensor gain of 0dB, but adjustments were needed if the weather was cloudy (gain increases from 5-10 dB). We therefore used the FlyCapture software to automatically optimize the sensor gain for each recording environment. Once the initial gain was set, it was kept fixed for the recording duration. The RAW file format should be linear with respect to incident light. Prior to recording, we confirmed this linearity by taking images of an X-Rite PANTONE ColorChecker board. We also quantified the barrel distortion of the camera lens using MATLAB’s Camera Calibrator app and the estimated lens parameters (6 parameters total) were used to correct for image distortion prior to analysis of the dataset.

#### Recording sites.

The dataset was collected across two sites to capture a variety of natural motion: the University of California, Berkeley campus in Berkeley, California and the Oakland Zoo in Oakland, California. The campus site provided motion from semi-urban scenes, resulting from foliage, pedestrians, and vehicles, while the zoo allowed for capturing scenes with animals. At each of these sites, a set of 4-minute movies were recorded at unique locations for a total of around 3 hours of footage (24 movies at the campus and 25 movies at the zoo). The recording camera was always mounted on a stationary weight-stabilized tripod. For practical reasons, the methods for choosing recording locations differed slightly for the two sites. At the campus site, random 2D coordinates were generated and placed on a map of the campus. Filming locations corresponded as closely as possible to these coordinates, allowing for slight adjustments to stay on sidewalks and lawns. At the zoo, enclosures with easily visible animals and no obstruction (e.g., glass walls, netting) were chosen to be filmed. For all locations, the camera direction was set by subjectively observing the direction in which the most visual motion was observed at the time of recording. A third site was initially selected in a nearby park, however, the video quality obtained from this site was low so it was excluded from analysis.

#### Motion estimation.

**Fig 1:**
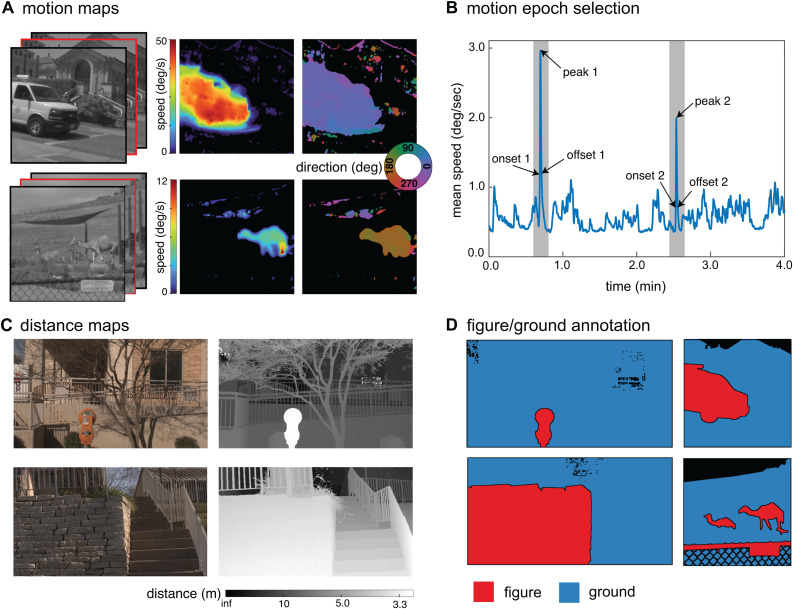
Motion maps and distance maps with figure-ground annotations were generated for analysis. **A)** A dataset of motion maps was created by applying motion estimation to grayscale movies (left), yielding estimates of motion speed (left) and direction (right). The red outline indicates the central frame for which motion was estimated. **B)** Epochs of visual motion for analysis were sampled from each 4-minute-long movie by selecting two local maxima in the mean speed and then sampling the frames associated with the peak, onset, and offset of motion. **C)** RGB images (left) and distance maps (right) were obtained from the dataset described in [32]. **D)** Figure region (red) and ground region (blue) annotations of selected images from the distance and motion map datasets were performed by professional human annotators. Black pixels indicate excluded sky regions and figure-ground boundaries.

The recordings were used to estimate the speed and direction of motion at each pixel in selected epochs of activity (Fig 1A). Motion estimation was performed using the MATLAB implementation of the deformable image registration algorithm described in Vishnevsky et al. (imregdeform) [33]. This method was selected due to its robustness to changes in scene illumination, ability to capture sharp motion edges, and capacity to simultaneously capture a wide range of speeds within a scene. As a sanity check, motion estimation was also performed using a standard algorithm for local optical flow based on differential motion estimation (the Farneback algorithm) and the global speed and direction results were overall similar but noisier so here we report the results from the more robust algorithm. To reduce errors due to pixel intensity noise, we smoothed the data slightly in both space and time prior to motion estimation. First, each frame was spatially median filtered with a 3x3 pixel filter. Next, we selected a target frame (f), computed the displacement field from the previous frame (f-1) and to the next frame (f + 1), and averaged the results together. Each frame pair was histogram equalized and then histogram-matched to optimize performance. Component motion estimates in the horizontal and vertical directions were converted to speed and motion direction. Motion direction is reported in degrees with 0 corresponding to rightward motion, 90 upward, 180 leftward, and 270 downward. While this method generally produced high-quality flow fields, manual inspection revealed consistent and significant motion noise in large featureless regions where the optimization is poorly constrained, most notably in regions of the sky. Sky regions were therefore excluded from analysis. Lastly, we measured the noise floor of the motion estimation algorithm by applying the same algorithm to sets of identical frames (i.e., frames for which the ground truth motion was zero). Across a set of samples from both recording sites, the maximum spurious motion speed was 0.14 pixels/frame (0.67 deg/s) and the 99th percentile was 0.02 pixels/frame (0.096 deg/s). We therefore conservatively limit our analysis to speeds that exceed 0.5 deg/s. The motion above this threshold likely still includes some spurious motion (see regions of detected motion around the background buildings in Fig 1A, upper row), however, higher thresholds appeared to remove legitimate slow speed motion.

#### Frame selection.

Two epochs of visual motion were identified in each of the movies for further analysis. Epochs were selected by first running a coarse calculation of the motion estimation between all frame pairs in each entire 4-minute movie. The mean optic flow speed across all pixels was used to estimate the amount of visual motion in each frame (Fig 1B). A Gaussian averaging window with a standard deviation of 120 frames was applied to smooth out the mean speed estimates. The two frames with the highest mean speed were then selected for analysis. These peak frames were forced to have a minimum separation of 3 s to ensure that distinct motion events were captured. Some frames were manually rejected and replaced with the next-highest mean speed if the majority of the motion stemmed from incidental camera movement (e.g., jostling of the tripod) or from spurious visual motion (e.g., in the sky). Once the two peak epoch frames were selected, frames representing the onset and offset of each motion epoch were then chosen. The frame with mean speed closest to 50% of the peak speed in the 2.5 s window before the peak frame was selected for the onset of the motion event; similarly, the frame with the mean speed closest to 50% of the peak speed in the 2.5 s window after the peak frame was chosen as the offset. Altogether, these three frames define points of interest in one epoch of visual motion (motion onset, motion peak, and motion offset). Using this method, six frames were selected for analysis from each movie. Motion estimation was then recomputed at full resolution for each of these frames. The motion patterns (both speed and direction) were ultimately highly similar across the onset, peak, and offset of motion, so we report the results aggregated across all frames.

### Distance map dataset

The distance map dataset was derived from a publicly available natural scenes database, for which the methods are described in detail elsewhere [32]. This database constitutes color images and distance maps captured at 98 locations around the campus of the University of Texas at Austin in Austin, Texas. Relevant information is briefly summarized here.

#### Recording device.

A custom-built robotic gantry was used to capture images (recorded with a Nikon D700 camera) and distance maps (recorded with Riegl VZ-400 3D laser range scanner) from the same viewpoint, enabling pixel level co-registration of image and distance information (Fig 1C). The images contain 3 color channels (red, green, and blue) with 14 bits per channel (linear and uncompressed). After cropping to minimize lens distortion effects, the final image resolution is 1920 x 1080 pixels, covering a field of view of 35 deg x 20 deg (~0.02 deg/pixel). While the original dataset consists of stereo pairs, here we limit our analysis to a single image/distance map per site (the right image from the original stereo pair). The range scanner uses time-of-flight laser sensing to provide distance measurements with a precision of +/- 5 mm, and is able to record distances between approximately 2 m and 200 m from the device. In practice, the scanner was positioned at each site such that the minimum object distance was approximately 3 m. Off-line processing was used to sample these distances at each image pixel location.

#### Recording sites.

The visual content in this dataset includes a mixture of man-made structures (e.g., buildings, signs, roads, cars), natural content (e.g., trees, bushes, lawns), and people. This content was similar to the content in the motion map dataset, particularly from the campus setting. The documentation indicates that recordings were taken at each site with an approximately earth-parallel visual axis at 1.7 m above the ground but otherwise does not state how the specific recording locations on the campus were selected.

### Figure ground annotation

A professional image annotation company (BUNCH) was employed to generate figure labels for both the motion map and distance map datasets. This approach was selected to ensure high-quality annotation borders and segmentations that reflect human interpretations of the scenes. For annotation of the motion map dataset, human annotators were instructed to view a 30-s long movie clip encompassing each epoch before beginning the annotation. The movie clip served to provide context to the still image to be annotated. Annotations were performed on the still images alone (i.e., the annotators were not provided with distance maps or motion maps to annotate). All images were gamma-corrected for viewing on a conventional display prior to annotation. In each image, annotators were instructed to identify 1–3 primary figure regions, which were defined as the “part(s) you would consider to be the main object(s) you’re looking at in the scene.” Aggregates of objects (e.g., a crowd of people, a cluster of leaves) were allowed to be considered as one figure as long as they were sufficiently close together. Once figures were identified, annotators used software tools to label all pixels within their selected figures. When possible, regions designated as figures were also specified in terms of a predetermined list of subcategories (e.g., person, bike, building, lawn). Annotators were instructed to label all other pixels in the images as either the remaining “ground regions” (i.e., surrounding area) or sky. A separate label was designated for sky so that sky pixels could be excluded from analysis. In the distance map dataset, the excluded sky regions constituted 0.6% of all pixels and in the motion map dataset it was 6.5%. However, in practice the number of actual sky pixels was higher because they were challenging to individually annotate. Example annotations are shown in Fig 1D.

### Data quality screening

Each motion map, distance map and annotation was manually reviewed for quality. For the motion map dataset, of the 98 motion epochs identified, 12 were excluded due to data quality issues (e.g., motion blur, registration errors, poor annotation alignment), resulting in 86 motion epochs for analysis (totaling 258 motion maps because each epoch included 3 clips representing the onset, peak and offset of motion). For the distance map dataset, of the original 98 recording sites 6 were excluded due to similar data quality issues. Fourteen additional exclusions were made based on a pilot study in which a cohort of human observers performed manual annotations in the lab, based on the observation that observers could not reliably identify specific figure/ground regions. Most of these excluded images contained heavy vegetation (e.g., bushes, plants, etc.) that was tightly packed together, making the boundaries unclear or ambiguous. These exclusions resulted in 78 scenes for our distance map analysis. In the final datasets, the overall alignment between the figure labels and the scene content was high quality. However, to avoid cross contamination when computing visual statistics of figure and ground regions, we excluded any points within a 5-pixel buffer around all annotation borders from analysis.

### Receptive field simulation

#### Spatial sampling.

**Fig 2:**
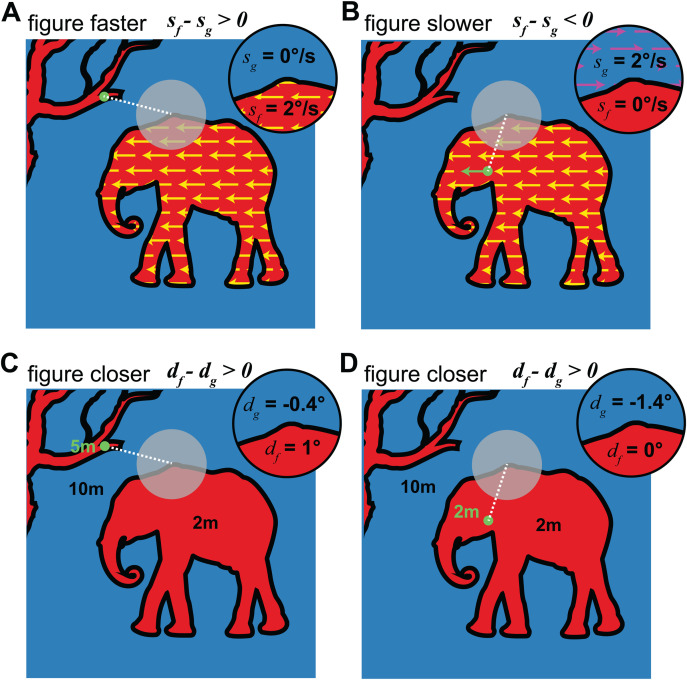
Simulated receptive fields (sRFs) and fixation points were used to sample from both datasets. This figure illustrates these sRFs and how the associated fixation point can affect retinal speed and disparity (it is not a real scene from our datasets). After an sRF (gray circles) was identified, we assigned a random fixation point based on the scene’s saliency map (green circles) at the appropriate eccentricity (white dashed lines). A,B) For the motion maps, the calculated motion vector at the point of fixation was subtracted from the motion at all other points to simulate retinal motion (inset). In this example, the annotated figure region is moving leftward and the rest of the scene is stationary. Panel A illustrates the average speeds in the resulting figure and ground regions (s_f_ and s_g_) for a stationary fixation point (such that the figure region speed is faster) and panel B illustrates the same for a moving fixation point on the figure (such that the ground region speed is faster). C,D) For the distance map dataset, the calculated distance at the point of fixation was used to determine the binocular retinal disparity. In this example, the annotated figure region is closer than the rest of the scene. Panel C illustrates average disparities in the resulting figure and ground regions (d_f_ and d_g_) for a far fixation point and panel D illustrates the same for a near fixation point on the figure. In both cases, the figure region has a more positive disparity than the ground. Images are modified from artwork obtained on Pixabay.

To examine local statistics, circular regions on par with the size of MT receptive fields were sampled from both the motion map and distance map datasets. These simulated receptive fields (sRFs) were of four diameters (2.5, 5, 10, and 15 deg), which correspond to the receptive field sizes of MT neurons associated with eccentricities in the parafoveal and peripheral visual field, as the receptive field sizes of MT neurons roughly match their eccentricities [34,35]. SRFs smaller than 2.5 deg were impractical to simulate due to the limited spatial resolution of the datasets, and larger ones were impractical due to the field of view of the cameras used to create each dataset. We generated 800 sRFs from the motion map dataset and 800 from the distance map dataset (200 of each sRF size). For each sRF, a random scene was first selected, and then a random position in the scene was selected for the center of the sRF (in Fig 2, gray circles illustrate sRF placements). SRFs were discarded if more than 50% of the pixels contained missing measurements (e.g., sky, motion below threshold). We aimed to examine the statistics of visual features at figure-ground borders, so samples were also discarded if they did not contain at least 25% pixels designated as figure and 25% designated as ground. No restrictions were applied to the number of distinct figure regions within the sRF. Finally, for each sRF we selected a putative fixation point in order to simulate the effects of gaze (Fig 2 green dots), and sRFs without a valid fixation point at the appropriate eccentricity were also discarded. This process was repeated until the desired number of samples was obtained for each sRF size.

#### Simulating Gaze.

The motion and distance of points in the world can translate to different patterns within a receptive field, depending on the gaze position and movement of the eyes. Thus, we implemented a simulation of gaze that takes into account visual saliency, visual tracking (smooth pursuit), and vergence eye movements. Saliency mapping algorithms can be used to predict where observers will fixate on a given image, typically incorporating visual elements such as contrast, feature uniqueness, and visual task [36]. There are many algorithms for computing visual saliency maps. We selected the bottom-up saliency estimation approach described in Zhang et al. [37], due to its incorporation of biologically-inspired operations and natural visual statistics [38,37]. Bottom-up saliency was computed based on pixel intensity values, and therefore did not incorporate potential effects of visual motion or distance of points on visual saliency, although motion and distance may also affect the probability that a point is fixated [39]. For each image in the motion map and distance map datasets, we selected the half of the pixels with the highest visual saliency as plausible fixation points (i.e., the upper half of a median split). Once a fixation point was selected for an sRF, we then used the motion and distance of this point to model eye movements.

For the motion analysis, we assumed the observer was making a smooth pursuit eye movement to track this point. We therefore subtracted the motion vector at fixation from the motion at each point within the sRF, obtaining an estimate of retinal motion. If the fixation fell on a point with speed below detection threshold, we assumed that fixation was not moving. Thus, for example, if the fixation fell on a static background, a moving object would result in the figure region having faster retinal motion (*s*_*f*_) than the ground region (*s*_*g*_) (Fig 2A). However, if the fixation point fell on a salient moving object, the eye movement could stabilize the motion in the figure region of the sRF, and result in slower retinal motion of the figure as compared to the ground (Fig 2B).

For the distance analysis, we assumed the observer was making a vergence eye movement to fixate this point (i.e., disconjugately rotating their left and right eyes so that the point fell on both foveas). We then computed the retinal binocular disparity of each point in the sRF accordingly – the angular difference in the retinal eccentricity in the left and right eyes in visual degrees. We focus on horizontal disparity, because tuning for horizontal binocular disparity is an important way in which depth-selective neurons encode distance information, and it scales lawfully with relative distance in units of diopters (inverse meters) [40,22]. Specifically, for each pixel in the scene, we obtained the three-dimensional coordinates with respect to the laser range scanner (P = [*x y z*]). We assumed that the left and right eyes of the observer were symmetrically offset from the scene origin along the horizontal axis by half of the interocular separation (*s*). We could then specify each scene point as a vector in left and right eye coordinates (P_R_ = [*x*_R_ *y z*], P_L_ = [*x*_L_ *y z*], where *x*_R_ = (0.5*s*)*-x* and *x*_L_* = -*(0.5*s*)*-x*). If the 3D coordinates of the fixated point are given by [***x y z***], then the horizontal angular eccentricity of each other point is: *θ*_*R,L*_ = atan(***x***_R,L_/***z***) - atan(*x*_R,L_/*z*). Finally, the binocular disparity is the difference between these two eccentricities (*d* = *θ*_*L*_ *- θ*_*R*_). We assumed an interpupillary separation of 6.2 cm and applied this calculation to each point in the sRF. We use the convention that crossed disparities, indicating points nearer than fixation, are positive and uncrossed disparities, indicating points farther than fixation, are negative. Importantly, while the amount and sign of the disparity relative to fixation may flip, points that are closer in a scene will always have a positive disparity difference from farther points, regardless of the vergence angle of the eyes. For example, if the disparity of a figure region (*d*_*f*_) is nearer than the disparity of a ground region (*d*_*g*_), the relative disparity will be positive regardless of the fixation point (Fig 2C and 2D). The original dataset also included camera stereopairs but we do not use these for our calculation of disparity. Using the stereopairs to compute angular disparities would require applying a computational stereo-matching algorithm (to identify corresponding points), which would produce noisier results than using the direct distance measurements from the laser.

Across these gaze simulations, the mean simulated pursuit speed was 7.8 deg/s (median = 0.7 deg/s) and the mean simulated vergence distance was 19.3 m (median = 11.8 m). Although the saliency model used to simulate fixations was independent of the figure/ground labels, the model had a slight bias to select fixations from figure regions: out of all pixels annotated as figures, 52% were also putative fixations. One element that is missing from this simulation is head motion and the associated VOR. While this is a gap in our current analysis, we think it is appropriate to leave investigation pertinent to self motion, for example during locomotion, for future investigations.

### Experimental design and statistical analyses

#### Global scene analysis.

We first examined the global statistics of motion speed, motion direction, and distance across the full scenes. This analysis was performed prior to performing the gaze simulations, so as to capture the statistics of the environment independent of the viewer. We computed the frequency distribution of these three features for all non-excluded pixels. We computed speed (the magnitude of the motion vector) in deg/s, direction in angular degrees, and distance in meters. Next, to examine overall differences in motion and distance between figure and the ground regions, we isolated pixels that were labeled as figures and pixels labeled as ground and computed the frequency distributions separately. We compared the figure and ground frequency distributions for speed and distance using rank sum tests (because that data deviated notably from normality). Rank sum effect sizes (*r*) were computed using the method described in [41]. We compared the statistics for motion direction between figure and ground regions using the Kuiper test, which tests for differences between a pair of circular distributions. We also used Rayleigh tests to assess whether each motion direction distribution differed significantly from uniform [42]. For all statistical tests, we use a threshold of *p* < 0.05 to determine significance.

#### sRF analysis.

For each sRF, which included spatial sampling and gaze simulations, we computed the average retinal motion speed, motion direction circular variance (as a measure of coherence), and disparity within the figure region and the ground region (that is, the remaining non-figure pixels that fell within the sRF). We then computed the difference between these two regions (figure-ground difference). MT neurons are thought to code for motion speed on a log scale [43], so we conducted our speed analysis on the log (base 10) of speed in deg/s. Distributions were approximately normal, so t-tests were used to examine whether the figure-ground differences deviated significantly from zero (that is, was the speed, coherence, or disparity of the figure regions within the sRFs different from the ground regions). We report *p* values; however, since *p* values are influenced by the number of simulations that were run we also report effect sizes as computed with Cohen’s D. One-way ANOVAs were used to examine if the figure-ground differences varied as a function of sRF eccentricity, with effect sizes reported as *η*^*2*^*.* Follow-up pairwise comparisons were performed using the Tukey HSD method. We also assessed the proportion of figure regions that were faster, more coherent, and nearer than the ground regions within the sRFs, and we report the overall proportion and the 95% binomial confidence intervals. Lastly, we examined how the figure-ground probability ratio of each feature varied continuously as a function of relative speed, motion direction, and disparity within the sRFs. To determine these ratios, we first quantified the frequency distributions of relative motion speed, motion direction, and disparity in 50 linearly-spaced histogram bins. The distributions for relative speed and motion direction were computed in normalized units. For speed, we normalized the speeds within each sRF to range from 0-1, excluding points above/below the 1st and 99th quantiles so as to be robust to outliers. For motion direction, the distribution of motion directions was rotated to align the most frequent direction within the sRF as 0 deg. Since binocular disparity is already scaled relative to the fixation distance, no additional normalization was used for this feature. Histogram frequencies were then averaged across all sRFs. We converted these frequencies to probability densities and computed the ratio between the figure and ground probabilities at each relative speed, motion direction, and disparity. We bootstrapped 95% confidence intervals for each ratio. The conversion to probability density assumes that there are no systematic differences in the sizes of figure and ground regions that fall within a given receptive field, which is supported by the data: the mean percentage of each sRF that was labeled figure and ground was 44% and 45% respectively in the motion map dataset and 45% and 50% in the distance map dataset (recall that pixels right at figure-ground borders were excluded from analyses, which is why these values do not add up to 100%). However, there were fewer pixels with valid motion measurements in the sRF ground regions due to our speed threshold, so the probability ratio provides a comparison of figure and ground features of the valid pixels only.

## Results

### Globally, figures tend to move faster, move more horizontally, and be nearer than their surroundings

**Fig 3:**
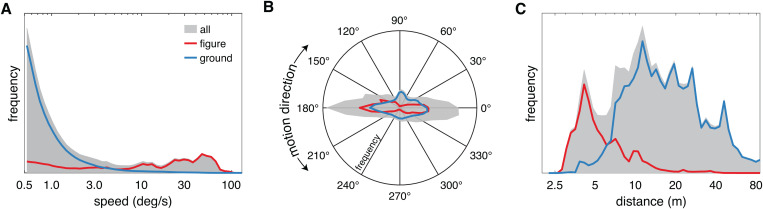
Globally, figures tend to move faster, move more horizontally, and be nearer than their surroundings. **(A)** The overall frequency distribution of all speeds (gray shading) is shown for the motion map data set, excluding motion below 0.5 deg/s. Red and blue lines show the distributions separately for pixels labeled as figure and ground, respectively. **(B, C)** The overall frequency distribution of motion directions and scene distances, as well as the figure and ground distributions, are plotted in the same manner as in A.

Before examining statistical features within simulated receptive fields, we start by characterizing global frequency statistics of world motion and distance in our datasets. With respect to motion, we found that speed in the scenes was skewed towards slower speeds. Across all scenes, 69.7% of the estimated motion was at or below 0.5 deg/s – that is, the majority of visible content was stationary or slow-moving. Of the remaining estimates, the median speed was 1.2 deg/s (Fig 3A gray shading). This pattern is consistent with previous studies of natural motion statistics [44,45]. However, the speed distribution in our dataset was also slightly bimodal, with a second smaller peak at relatively fast speeds (~45 deg/s). The peak at fast speeds was caused by moving figures, whereas the slower peak was dominated by the ground regions but also contained stationary figures (Fig 3A red and blue lines).

Figures in the motion map dataset tended to be people, cars, small infrastructure, and animals (representing 15.7% of the valid pixel labels). The mixture of animate and inanimate figure classes is likely the cause of the broad distribution of figure speeds. The area surrounding the figures, however, was not completely stationary: inspection of the movies suggested that ground motion tended to be caused by wind-blown vegetation and animate objects that were not labeled as figures (e.g., people far from the camera), as well as moving shadows. On average, for the above threshold motions we found that the figures tended to move faster (median = 21.2 deg/s) than the ground regions (median = 0.8 deg/s), and this difference was statistically significant (*z* = 5219, *r* = 0.33, *p*<<0.001). However, we also observed notable overlap in the figure and ground speed distributions.

Motion direction had a tendency towards horizontal motion (Fig 3B gray shading) and this horizontal dominance was most visually pronounced in the figures (Fig 3B red line) as compared to the ground regions (Fig 3B blue line). This difference likely reflects the tendency of animate figures to move orthogonal to gravity along the ground plane, which will favor horizontal retinal motion for an observer also standing on the ground. Consistent with these observations, both the figure (*z* = 123900, *p*<<0.001) and ground direction distributions (*z* = 86063, *p*<<0.001) differed significantly from being uniform and the distributions were also significantly different from each other (*k* = 7.00x10^13^, *p* = 0.001).

The distribution of distances was also slightly bimodal, with a median of 12.9 m (Fig 3C gray shading). This pattern is generally consistent with previous measurements of natural scene geometry, and the relatively far median reflects the fact that this dataset was focused on views without much content near the camera (i.e., no objects closer than 3 m) [46,39,47]. Figures in the distance map dataset represented 23% of the valid pixel labels, and tended to be buildings and small infrastructure. On average, we found that the figures (Fig 3C red line, median = 4.9 m) tended to be nearer than the ground regions (Fig 3C blue line, median = 16.8 m), and this difference was statistically significant (*z* = -7725, *r* = 0.62, *p*<<0.001). However, it was not always the case that a figure was nearer than the surroundings. For example, regions of lawns and pathways were visible in front of figures like buildings. Indeed, as for the motion distributions there was substantial overlap in the figure and ground distance distributions.

In summary, the global statistics suggest that there is a tendency for visual figures to move faster and in a more dominantly horizontal direction than the ground regions, as well as to be nearer in distance. Given the strength of these patterns, it seems likely that visual input at the level of neuronal receptive fields will exhibit robust statistical regularities. However, these global patterns may be disrupted or altered by local figure-ground relationships as well as the effects of gaze direction and eye movements. We explore our simulation of the resulting receptive field statistics in the next sections.

### Within simulated receptive fields, figure regions tend to move faster than the ground regions

**Fig 4:**
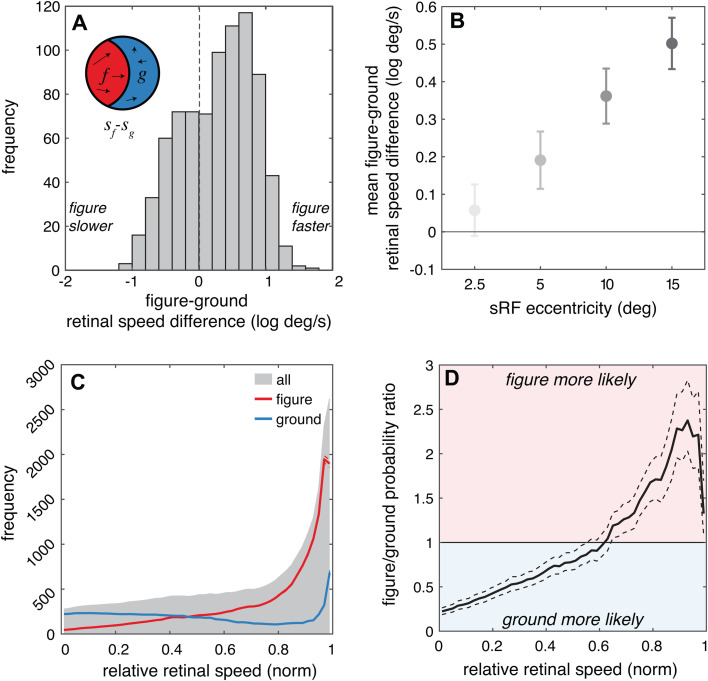
Within sRFs, figure regions tend to move faster than the ground regions. **(A)** The frequency distribution of speed differences between figure (f) and ground (g) regions in log deg/s across all sRFs is plotted. Positive values indicate sRFs in which the average figure moves faster than the average speed in the ground. **(B)** The average and 95% confidence intervals for these figure-ground speed differences are plotted separately for each sRF eccentricity. **(C)** For relative speed, we show the average frequency across all points in the sRFs (gray shading), and separately for the figure regions and ground regions (red and blue lines, respectively). **(D)** The mean figure and ground results from panel C are plotted as a probability ratio as a function of relative speed. Values greater than 1 (red background) indicate that points are more likely to be in figure regions and values less than 1 (blue background) indicate that points are more likely to be in ground regions. In C and D, dashed lines indicate 95% confidence intervals, but in some panels the intervals are barely wider than the line thickness.

We start by examining how the world speed statistics translate to local regions comparable to MT receptive fields (the sRFs). Consistent with the global statistics, we found that points associated with the figure within an sRF tended to have faster retinal speeds than the ground within that sRF. The mean figure-ground retinal speed difference (*s*_*f*_ - *s*_*g*_) within an sRF was 0.28 log[deg/s] (5.7 deg/s) which was significantly greater than zero (*t*(799)=14.5, *p*<<0.001, *D* = 0.51) (Fig 4A). Across all sRFs, the average figure region speed was faster than the average ground region speed 68% of the time (CI: 65%-71%).

There is one notable situation in which it seems unlikely for figure regions to consistently cause faster retinal motion than ground regions: when the eyes are tracking the same visual figure that falls within the receptive field (see Fig 2B). In this case, the eye movement would stabilize the figure motion on the retina, resulting in a bias towards slower speeds in figure regions. We therefore hypothesized that a slower figure bias might be likely to occur in sRFs nearer to the fovea, because the small eccentricity of the sRF would increase the likelihood that the nearby salient features were part of the same object that comprised the figure region in the sRF. Indeed, we found that the average figure-ground speed difference varied significantly as a function of sRF eccentricity (Fig 4B, *F*(3,796) = 28.2, *p* << 0.001, *η*^*2*^ = 0.1). We did not observe a slow speed figure bias at any eccentricity, however, the figure-ground speed difference was near zero for the lowest eccentricity sRFs and increased significantly with increasing eccentricity. All pairwise comparisons were statistically significant (Table 1). For the lowest eccentricity sRFs, the average figure speed was faster than the average ground speed only 52% of the time (CI: 44%-59%), whereas for the highest eccentricity sRFs, the average figure speed was faster than the average ground speed 82% of the time (CI: 76%-87%).

**Table 1:** Pairwise t-tests comparing figure-ground speed differences at each sRF eccentricity.

Ecc. 1	Ecc. 2	Mean diff. log[deg/s]	CI	p	Cohen’s D
2.5°	5°	-0.13	(-0.27, 0.00)	0.050	0.25
2.5°	10°	-0.30	(-0.44, -0.17)	<0.001	0.59
2.5°	15°	-0.44	(-0.58, -0.31)	<0.001	0.90
5°	10°	-0.17	(-0.30, -0.04)	0.006	0.32
5°	15°	-0.31	(-0.44, -0.18)	<0.001	0.60
10°	15°	-0.14	(-0.27, -0.01)	0.032	0.28

P values were computed using the Tukey HSD method. Ecc = eccentricity, Mean diff = mean difference between groups, CI = 95% confidence interval of this difference.

In addition to these average speed differences, we also examined how the probability that a given point corresponded to the figure versus the ground varied from the slowest point to the fastest point within the sRF. First, we examined the frequency distribution of relative speeds in the figure regions and ground regions (Fig 4C, red and blue lines). This analysis is analogous to the global speed analysis (Fig 3A) but it focuses on the relative speeds with the sRFs, incorporating the effects of spatial sampling and gaze dynamics. Similar to the global speed distributions, the distribution of figure speeds was biased towards faster speeds than the ground speed distribution (the gray shading shows the frequency distribution of all points within an sRF). Accordingly, the ratio of the figure/ground probabilities varied: slow speeds were more likely to correspond to the ground regions and faster speeds to figure regions (Fig 4D). A slight reduction in the figure/ground probability ratio is observed at the highest speeds, which occurs because the frequency distribution of figure speeds peaks slightly below the maximum relative speed in the sRFs on average.

### Within simulated receptive fields, figure regions tend to move slightly more coherently than the ground regions

**Fig 5:**
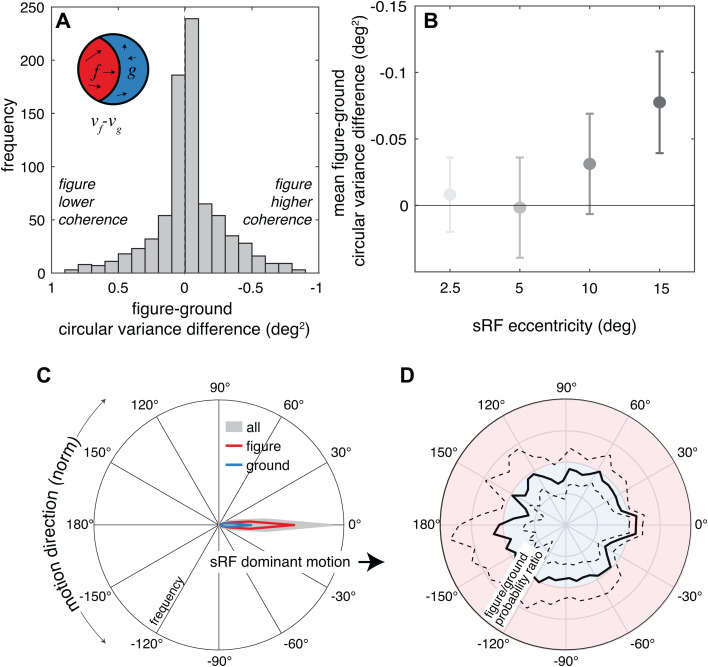
Within sRFs, figure regions tend to move more coherently than the ground regions. **(A)** The frequency distribution of differences in circular variance between figure and ground is plotted as a measure of coherence difference. Positive values indicate lower coherence in the figure region and negative values indicate higher coherence in the figure region. Note that the x axis is flipped. **(B)** The average and 95% confidence intervals for these figure-ground variance differences are plotted separately for each sRF eccentricity. **(C)** For relative direction, we show the frequency across all points in the sRFs (gray shading), and separately for the figure regions and ground regions (red and blue lines, respectively). 0 deg corresponds to the dominant motion in each sRF. **(D)** The mean figure and ground results from panel C are plotted as a probability ratio as a function of relative motion direction. Values greater than 1 (red background) indicate that points are more likely to be in figure regions and values less than 1 (blue background) indicate that points are more likely to be in ground regions. In C and D, dashed lines indicate 95% confidence intervals, but in some panels the intervals are barely wider than the line thickness.

We also considered that the horizontal motion direction bias observed in the global scene analysis (Fig 3B) might cause figure regions to have more coherent motion directions than ground regions in sRFs. Thus, we compared the circular variance of the motion directions within figure regions (*v*_*f*_) and ground regions (*v*_*g*_). Consistent with our expectation, we found that the circular variance in figure regions tended to be slightly lower than the ground regions, suggesting more coherent figure motion (Fig 5A). The mean figure-ground difference in circular variance was -0.03 deg^2^ and significantly less than zero (*t*(799)=-3.2, *p* = 0.002, *D* = 0.11) – although the effect size was notably smaller than the one associated with figure-ground speed difference. Across all sRFs, the average figure region variance was lower than the average ground region variance 58% (CI: 54%-61%) of the time. We hypothesize that the lower coherence in ground regions results from a combination of complex motion patterns in background foliage as well as animate features that were not identified as figures, such as multiple people in the background walking in different directions. This coherence difference was again eccentricity dependent, with a larger difference associated with higher eccentricity sRFs (*F*(3,796) = 3.8, *p* = 0.010, *η*^*2*^ = 0.01), although the effect size was again smaller (Fig 5B). Pairwise comparisons between the highest eccentricity (15 deg) and the two lowest (2.5 and 5 deg) were both statistically significant (Table 2). For the lowest eccentricity sRFs, the average figure region variance was lower than the average ground variance only 49% of the time (CI: 42%-56%), whereas for the highest eccentricity sRFs, it was lower 71% of the time (CI: 64%-77%). This pattern, however, was not clearly reflected in the normalized direction histogram. As a reminder, normalized directions were computed by first determining the dominant (most frequent) direction in the sRF and then rotating the histogram to align the dominant direction at 0 deg. While there was a strong tendency for all motion directions to align with the dominant motion (Fig 5C, 0 deg), the figure-ground probability ratio was relatively isotropic across motion directions (Fig 5D).

**Table 2:** Pairwise t-tests comparing figure-ground circular variance differences at each sRF eccentricity.

Ecc. 1	Ecc. 2	Mean diff. deg^2^	CI	p	Cohen’s D
2.5°	5°	-0.01	(-0.08, 0.06)	0.976	0.04
2.5°	10°	0.02	(-0.04, 0.09)	0.814	0.10
2.5°	15°	0.07	(0.00, 0.14)	0.036	0.29
5°	10°	0.03	(-0.03, 0.10)	0.566	0.12
5°	15°	0.08	(0.01, 0.15)	0.011	0.29
10°	15°	0.05	(-0.02, 0.11)	0.272	0.17

P values were computed using the Tukey HSD method. Ecc = eccentricity, Mean diff = mean difference between groups, CI = 95% confidence interval of this difference.

Taken together, the speed and coherence results suggest distinct statistical regularities in natural motion of figure regions at the level of neuronal receptive fields, particularly in the peripheral visual field. The specific prediction of this simulation is that retinal speeds falling in receptive fields that are faster are more likely to be figures (although clearly figures may also be stationary or slow). When figures are moving, their motion is slightly more likely to move in a coherent direction, providing an additional motion-based cue for figure-ground segregation within local receptive fields.

### Within simulated receptive fields, figure regions tend to be nearer than the ground regions and have more positive binocular disparity

**Fig 6:**
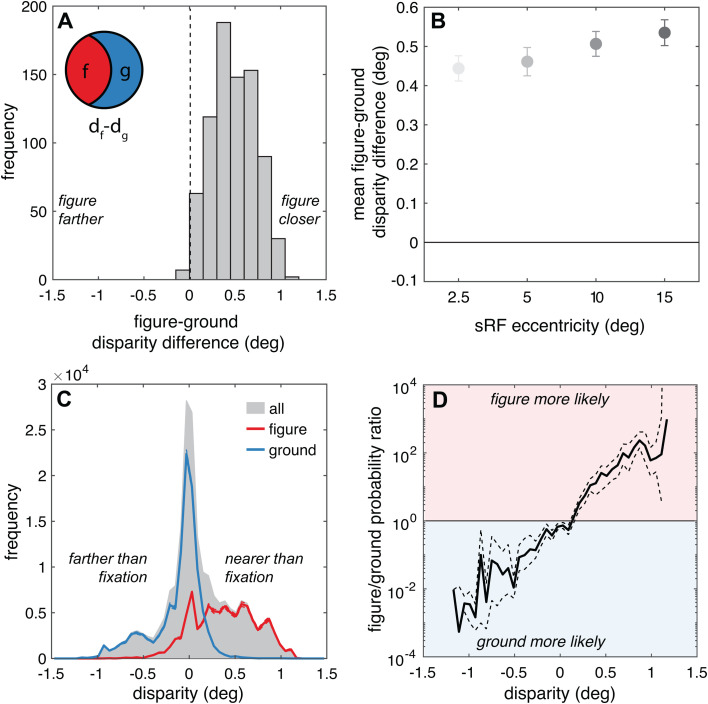
Within sRFs, figure regions tend to be nearer than the ground regions and have more positive (nearer) binocular disparity. **(A)** The frequency distribution of relative disparity between figure and ground across all sRFs is plotted. Positive values indicate sRFs in which the average figure distance is nearer than the average distance in the ground. **(B)** The average and 95% confidence intervals for these figure-ground disparity differences are plotted separately for each sRF eccentricity. **(C)** The frequency of binocular disparities is presented for figure regions and ground regions (red and blue lines, respectively). Gray shading indicates the probability across all points in the sRFs. **(D)** The mean figure and ground results from panel C are plotted as a probability ratio as a function of binocular disparity. Values greater than 1 (red background) indicate that points are more likely to be in figure regions and values less than 1 (blue background) indicate that points are more likely to be in ground regions. In C and D, dashed lines indicate 95% confidence intervals, but in some panels the intervals are barely wider than the line thickness.

We next examined how the scene-wide distance statistics translate to binocular disparities in the sRFs. Consistent with the global statistics, the figure regions tended to be nearer than the surrounding ground region within an sRF, and therefore have more near (positive) binocular disparity. The mean relative disparity between figure and ground regions was 0.49 deg, reflecting the fact that figure regions were significantly and consistently closer than ground regions (*t*(799)=56.7, *p*<<0.001, *D* = 2.00) (Fig 6A). As with the motion statistics, this disparity difference was significantly eccentricity-dependent, with larger eccentricities associated with larger disparity differences *(F*(3,796)=6.0, *p*<<0.001, *η*^*2*^ = 0.02) (Fig 6B). However, the effect size associated with eccentricity was quite small and the disparity difference was notably positive across all eccentricities. This difference increased slightly but significantly with increasing eccentricity (from 0.44 to 0.54 deg from the smallest to the largest eccentricity), with most pairwise comparisons statistically significant (Table 3). This makes sense – if a given point is closer than another point in space, the relative distance relationship, and therefore relative disparity, is not affected by the vergence state of the eyes. At each of the sRF eccentricities, the average figure disparity was nearer than the average ground disparity 98–100% of the time.

**Table 3:** Pairwise t-tests comparing figure-ground disparity differences at each sRF eccentricity.

Ecc. 1	Ecc. 2	Mean diff. deg	CI	p	Cohen’s D
2.5°	5°	-0.02	(-0.08, 0.04)	0.886	0.07
2.5°	10°	-0.06	(-0.12, 0.00)	0.046	0.27
2.5°	15°	-0.09	(-0.15, -0.03)	<0.001	0.39
5°	10°	-0.05	(-0.11, 0.02)	0.239	0.18
5°	15°	-0.07	(-0.14, -0.01)	0.012	0.30
10°	15°	-0.03	(-0.09, 0.03)	0.635	0.12

P values were computed using the Tukey HSD method. Ecc = eccentricity, Mean diff = mean difference between groups, CI = 95% confidence interval of this difference.

The probability that a given point corresponded to the figure versus ground also varied from crossed to uncrossed disparities. Fig 6C shows the average frequency distribution of figure regions (red line) as compared to the ground regions (blue line) across disparity (the gray shading again shows the distribution of all points within an sRF). The figures were notably biased towards crossed, positive disparities (nearer distances), while the ground regions were biased towards uncrossed, negative disparities (farther distances). This pattern is consistent with the global distance statistics, which showed that figures tend to be nearer (Fig 3C). As such, the ratio of the figure/ground probability was highest for near points and reversed for far points (Fig 6D).

Overall, these results suggest a robust statistical regularity in natural retinal binocular disparities at the level of neuronal receptive fields that complements the natural motion statistics. When individual receptive fields are stimulated by surfaces at multiple distances at a figure-ground border, the closer surfaces are more likely to be figures, which translates to a strong disparity-based figure-ground cue: points with nearer disparity are more likely to correspond to visual figures. But unlike the speed statistics, this pattern was present and similar in magnitude across all eccentricities.

## Discussion

### Relationship with neurophysiological recordings: Motion

When presented with stimuli moving at two different speeds within their receptive fields, individual MT neurons have response biases toward the faster component across a broad range of stimulus speeds [27,26]. Specifically, neuronal responses to these “bi-speed” stimuli are well-explained as a weighted sum of the responses to the component speeds, with a higher weight given to the faster speed component. Because our results suggest that motion associated with figures in receptive fields tends to be faster than motion associated with ground regions, a neural representation that favors stimulus elements with faster speeds may help to prioritize the representation of visual figures.

**Fig 7:**
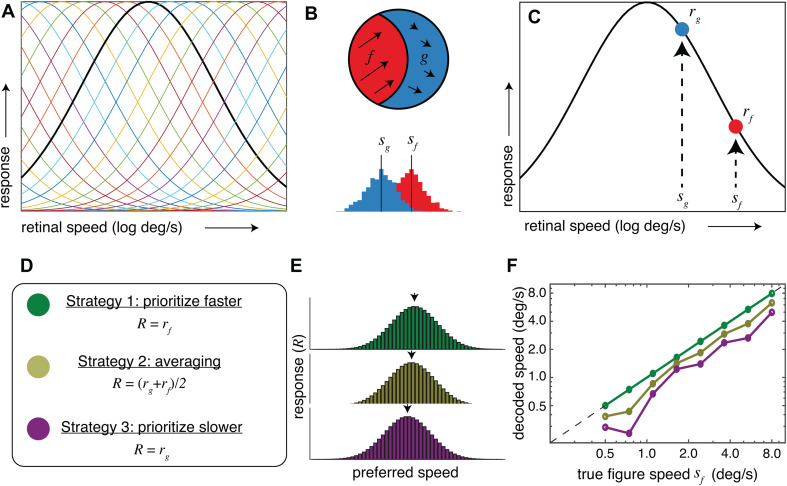
Toy example of neural population responses to figure-ground borders with faster figure speeds. **A)** Each line represents a neuron’s tuning curve within a population of neurons broadly tuned for stimulus speed. One example tuning curve is indicated with a thicker line. We assume the receptive fields of these neurons are spatially overlapping. Our model included 50 neurons with Gaussian tuning curves of standard deviation equal to 1 log[deg/s] and with means uniformly spaced in log speed. **B)** We consider a stimulus within this spatial location that contains a figure region (f) and a ground region (g). An illustrative histogram indicates that the average speed in the figure region (s_f_) is faster than the average speed in the ground (s_g_). **C)** An individual neuron in the neural population is shown – when stimulated by a figure-ground border this neuron will receive input consistent with both speeds (s_f_ and s_g_). In isolation, these speeds would elicit response rates of r_f_ and r_g_, respectively. **D)** We considered 3 possible strategies to determine the neural response to bi-speed input (R): prioritizing the faster speed such that the bi-speed response matches the response to the faster stimulus, averaging, and prioritizing the slower speed such that the bi-speed response matches the response to the slower stimulus. **E)** A simple decoding strategy was used to recover the stimulus speed from the population responses associated with each of these strategies: we computed the response-weighted average of each neuron’s preferred single stimulus speed. **F)** The decoded speed as a function of the speed of the figure region is plotted for eight example stimuli. For each stimulus, s_f_ was first selected and then s_g_ was set to be slower by a random scale factor ranging from 0.3 to 0.8. Line/marker colors correspond to the three strategies in panel **D.**

However, figure-ground segregation relies on populations of neurons, not individual ones. We thus performed a population-level simulation to explore how the interaction between individual neuronal response biases and figure motion statistics might play out at the level of a neural population. We start with a neural population with Gaussian tuning curves over log speed (Fig 7A). We assume that the receptive fields of the neurons in this population are spatially overlapping, such that together they encode the stimulus speed at a particular visual field location. We next consider a stimulus at this location that contains a figure region and a ground region (Fig 7B). The average speed in the figure region (*s*_*f*_) is faster than the average speed in the ground (*s*_*g*_) by some variable amount. Although there is also variation within each region, for simplicity we will just consider the averages. An individual neuron in the population (Fig 7C) will receive input consistent with both speeds (*s*_*f*_ and *s*_*g*_), which individually would elicit response rates of *r*_*f*_ and *r*_*g*_, respectively. We considered three possible strategies to determine the response of each individual neuron to the bi-speed input: prioritizing the faster speed, response averaging, and prioritizing the slower speed (Fig 7D). We implemented a simple population decoder (response-weighted average of all tuning preferences) for each strategy (Fig 7E). Fig 7F shows the decoded speed as a function of the speed of the figure region. The ‘prioritize faster’ strategy (green) – inspired by the empirical measurements from MT described above – reliably decodes the speed of the figure, whereas the averaging (yellow) and ‘prioritize slower’ (purple) strategies decode speeds that are slower than the figure. This example illustrates how natural statistics in figure-ground speed differences could be leveraged to prioritize the representation of the figure speed, although more sophisticated decoders may be able to also leverage these differences to recover both the figure and ground speeds (see [27]).

While this fast speed bias has been observed in MT neurons when the stimulus moved with a broad range of speeds from 1.25 - 40 deg/s, the MT responses to even faster stimulus speeds were observed to be closer to averaging or a slow speed bias [26,27]. We hypothesize that the fast speed regime might represent situations in which retinal motion is likely dominated by self-motion and the resulting motion parallax on the retinas, such that faster retinal speed is no longer a strong discriminating factor for figures. Indeed, our current motion statistics reflect situations in which retinal motion is overall slow (less than 20 of our 800 samples contained retinal speeds >20 deg/s in both the figure and the ground). In this regime, motion-based cues might particularly support figure-ground segregation, for example, to plan the next eye movement that tracks a new target of interest. Indeed, the majority of MT neurons prefer speeds less than 20 deg/s [43].

A similar bias has been observed on the basis of motion coherence: MT responses to two overlapping stimuli moving in different directions are biased to the component direction that has a higher motion coherence [28]. We also found that figure regions tend to contain more coherent retinal motion, but this pattern was weaker than the pattern observed for speed. We speculate that since both figure region motion (e.g., locomotion of animals and people) and ground motion (e.g., wind-blown vegetation) may contain good directional coherence in the world, the ultimate amount of coherence difference on the retinas is limited. Nonetheless, our results suggest that neuronal biases towards higher motion coherence may also support the representation of figural motion.

### Relationship with neurophysiological recordings: Disparity

A recent neurophysiological study suggests a similar connection between figure-ground distance statistics and MT responses to disparity, although the relationship is less direct. Chakrala et al. [29] found that MT neurons show a bias for one disparity over the other when presented with overlapping stimuli at two different disparities (“bi-distance” stimuli); however, the bias differed between animals. In one animal, the bias was always toward the nearer disparity regardless of the neuron’s disparity preference for single surfaces. In two animals, MT neurons were biased toward the surface that was most similar to the neuron’s preferred disparity [29]. Combined with previous findings that more neurons in MT prefer near disparities when stimulated by single surfaces (DeAngelis and Uka, 2003), a population level bias toward the preferred disparity could support the representation of near visual figures. However, a meta-analysis of disparity tuning in a different brain area (V1) suggested that population-level near-biases may be spurious [39]. Nonetheless, the figure-ground probability ratio for near disparities in natural scenes documented here is quite strong, so even a weak neural bias might be sufficient to support prioritization of figures. Additional investigation into both disparity tuning biases and visual statistics is needed to understand this potential connection more deeply.

### Effect of eye movements

In this study, we simulated gaze and assumed that the foveal stimulus was being tracked perfectly with a smooth pursuit eye movement. To the extent that these simulations deviate from real gaze patterns, they may result in biased estimates of visual statistics. For example, it has been shown that the speed of eye movements can lag substantially behind the speed of stimulus motion [48,49]. In some scenarios, the point falling on the fovea may not be pursued at all. To examine how robust our findings are to different gaze assumptions, we re-ran the sRF motion analyses assuming that the observer does not pursue the object at fixation and instead keeps the eyes stationary. That is, we simply removed the pursuit simulation. The key motion statistics were largely robust to this manipulation. With a stationary eye, figure motion on the retina was faster than the ground motion 95% of the time and more coherent 81% of the time (as compared to 68% and 57% with pursuit). This analysis suggests that the main effect of pursuit eye movements was to reduce the motion differences between figure and ground derived from world motion, rather than to amplify these differences. Interestingly, both the figure-ground speed and coherence differences tended to increase with eccentricity even without pursuit (speed: F(3,796) = 91.4, p < 0.001, η^2^ = 0.26, coherence: F(3,796) = 25.9, p < 0.001, η^2^ = 0.09). We hypothesize that receptive field size differences, on their own, may be sufficient to drive some of the eccentricity-dependent effects in Figs 4B and 5B, which may then be further enhanced via eye movements.

### Testable predictions for eccentricity-dependent neural differences

Our findings make new predictions about how neural responses to bi-speed and bi-distance stimuli should vary as a function of retinal eccentricity. We found that the figure-ground motion differences increased systematically with retinal eccentricity, but the magnitude of figure-ground disparity differences was relatively constant over eccentricity (Figs 4B, 5B, and 6B). If our working hypothesis that neural response biases are connected to figure-ground statistics is correct, then neurons should be more likely to prioritize fast speeds and coherent motion in peripheral and perifoveal vision as compared to foveal vision. Consistent with this hypothesis, the neurons that were observed to have a faster speed bias in Huang et al. [27] had receptive fields with an average eccentricity around 11 deg, which falls within the range where figure regions consistently tended to move faster. However, limited variability in the eccentricities from this dataset precluded a post hoc analysis to test for eccentricity-dependence. On the other hand, we would not expect to see a strong eccentricity-dependent effect for disparity-driven responses to bi-distance stimuli. Receptive fields tend to be smaller near the fovea, so it is also possible that neurons with foveal receptive fields are more likely to be stimulated by only figure regions or only ground regions, rather than a combination. If that is the case, we might expect foveal receptive fields to differ fundamentally in their coding of bi-speed and bi-distance stimuli. While neural recordings across a broad range of eccentricities and tuning properties can be challenging to obtain, these predictions highlight visual field eccentricities as a key element in the interaction between neural responses and figure-ground segregation during natural experience.

### Limitations

There is always a concern when measuring natural statistics that findings are influenced by the measurement devices, processing algorithms, and sampling biases. While we used calibrated equipment, avoided image compression and nonlinearities, and confirmed that motion findings were robust to different algorithms, caveats still remain. For example, some amount of the motion in our data may be due to sensor noise and spurious measurements from the sky. Also in both datasets, the choices of samples were made by experimenters: these samples therefore may not reflect overall statistics of natural experience. One way to address the sampling concern is to use first-person wearable capture methods [50,51,39]. Such methods would also address the limitations of our saliency model, which does not capture potential differences in salience for fast moving or nearby objects, and the lack of self motion in our data set. Notably, statistics captured in this way would include effects of motion parallax, enriching our understanding of how motion cues support figure-ground segregations in a broader set of behavioral contexts. Given that different animals likely experience different visual statistics, it would also be desirable to have data derived from the visual experience, behavior, and neural responses of the same animal. However, here we wanted to focus on high-quality measurements of motion and distance in the world, which required the use of equipment that was not amenable to wearable systems. In addition, the nature of this study required semantic segmentations with very precise boundaries between figure and ground regions; we thus decided to use a professional annotation company to generate the figure-ground labels. This constrains the number of annotators that were used for each individual frame. Because figure-ground judgments can vary amongst individuals (Shishikura et al., 2023; Yamane et al., 2020), this makes the annotations subject to the biases of the annotator. Ideally, several annotators would draw figure-ground designations for the same frame, and the results would be averaged across individuals.

## Conclusion

The statistical regularities found in this study, although intuitively sensible, are not a given. These findings therefore provide quantitative context for understanding neural coding principles that the visual system uses to adapt to the environment in essential tasks such as figure-ground segregation. By combining the statistical analysis of natural scenes with information about behavioral relevance, we can begin to parse out the visual cues that are pertinent to specific perceptual processes in natural environments and understand how these cues might map onto neural representations in specific brain areas.
